# *Acidithiobacillus ferrivorans* SS3 presents little RNA transcript response related to cold stress during growth at 8 °C suggesting it is a eurypsychrophile

**DOI:** 10.1007/s00792-016-0882-2

**Published:** 2016-10-25

**Authors:** Stephan Christel, Jimmy Fridlund, Elizabeth L. Watkin, Mark Dopson

**Affiliations:** 1 Centre for Ecology and Evolution in Microbial Model Systems (EEMiS), Linnaeus University, Kalmar, Sweden; 2 School of Biomedical Sciences, Curtin University, Perth, 6845 Australia

**Keywords:** Extremophile, Psychrophile, Cold acclimation, Compatible solutes, Metabolism, Electron transport

## Abstract

*Acidithiobacillus ferrivorans* is an acidophilic bacterium that represents a substantial proportion of the microbial community in a low temperature mining waste stream. Due to its ability to grow at temperatures below 15 °C, it has previously been classified as ‘psychrotolerant’. Low temperature-adapted microorganisms have strategies to grow at cold temperatures such as the production of cold acclimation proteins, DEAD/DEAH box helicases, and compatible solutes plus increasing their cellular membrane fluidity. However, little is known about *At. ferrivorans* adaptation strategies employed during culture at its temperature extremes. In this study, we report the transcriptomic response of *At. ferrivorans* SS3 to culture at 8 °C compared to 20 °C. Analysis revealed 373 differentially expressed genes of which, the majority were of unknown function. Only few changes in transcript counts of genes previously described to be cold adaptation genes were detected. Instead, cells cultured at cold (8 °C) altered the expression of a wide range of genes ascribed to functions in transcription, translation, and energy production. It is, therefore, suggested that a temperature of 8 °C imposed little cold stress on *At. ferrivorans,* underlining its adaptation to growth in the cold as well as suggesting it should be classified as a ‘eurypsychrophile’.

**Electronic supplementary material:**

The online version of this article (doi:10.1007/s00792-016-0882-2) contains supplementary material, which is available to authorized users.

## Introduction

Organisms referred to as extremophiles possess genetic traits allowing them to propagate in environments uninhabitable by the majority of microorganisms; be it due to unsuitable pH, temperature, salt or heavy metal concentrations, or other environmental factors (Cowan et al. 2015). The ability to thrive in extreme environments often makes these microorganisms excellent tools for biotechnological applications (reviewed in, e.g., Sarethy et al. 2011; Dalmaso et al. 2015; Siddiqui 2015). One application of extremophiles is for ‘biomining’, a term that describes the microbial promoted oxidation of insoluble metal sulfides to water soluble salts (Johnson 2014). During biomining, extremely acidophilic microorganisms (optimum growth pH ≤3) catalyze the chemical oxidation of metal sulfides by providing ferric iron via oxidation of ferrous iron and protons resulting from metabolism of the generated inorganic sulfur compounds (ISCs) (Bonnefoy and Holmes 2012; Dopson and Johnson 2012). Although biomining is responsible for up to 20 % of the worldwide copper production (Watling 2006), in Europe this biotechnology has only been implemented at an industrial scale at one site, the Sotkamo mine, Talvivaara, Finland (Riekkola-Vanhanen 2013). The large temperature differences present at the mine site (minimum air temperature in winter ~−30 °C) have resulted in the development of a microbial community containing species adapted to a range temperatures (Halinen et al. 2012).

In the past, ‘psychrotolerant’ microorganisms have been defined to exhibit optimal growth temperature above 15 °C and the ability to survive at temperatures below 0 °C while ‘psychrophiles’ have maximum growth rates below 10 °C (De Maayer et al. 2014). However, as growth rates increase with faster reaction kinetics at higher temperatures, this definition has little value to describe an organisms’ adaptation to its environment (Cavicchioli 2015). To solve this problem, the terms ‘eury-’ and ‘stenopsychrophile’ have been suggested to describe organisms with a wide and narrow temperature tolerance, respectively (Bakermans and Nealson 2004; Cavicchioli 2015). By establishing these designations, the term ‘psychrophile’ is appropriate to use for any organism indigenous to cold environments. Due to their size-related inability to insulate their cellular components, cold temperatures can challenge microorganisms by a number of factors including low nutrient diffusion rates, difficulties in protein folding and DNA supercoiling, and over-stabilization of mRNA and DNA secondary structures (D’Amico et al. 2006; Casanueva et al. 2010). To battle these adverse effects, psychrophilic microbes have developed a number of strategies. A major response to cold is the expression of cold shock proteins (CSPs), a loosely defined group of proteins of different function found up-regulated during sensing and, unlike the name suggests, also prolonged exposure to cold (Phadtare and Inouye 2004). However, not all CSPs are up-regulated at low temperature and they can have other functions not related to growth at low temperatures (Horn et al. 2007). Members of the CSPs also overlap with the group of cold-inducible proteins (CIPs), enzymes used by microbes to maintain cell integrity and metabolism during prolonged low temperature periods (Barria et al. 2013). Functions of both CSPs and CIPs are various and not limited to cold response, but include nucleic acid binding chaperones that melt cold-induced mRNA and DNA secondary structures, allowing ribosomes or RNA polymerases access to their substrate (D’Amico et al. 2006; Phadtare and Severinov 2010). Besides CSPs and CIPs, desaturases also provide a cold adaptation mechanism by introducing double bonds into fatty acid residues of existing membrane lipids and thereby, increasing membrane flexibility and restoring its semi-fluid state when it becomes too rigid (Chintalapati et al. 2004). Another strategy includes the production or assimilation of compatible solutes, small organic compounds that reduce osmotic stress induced by high ion concentrations in the liquid medium due to freezing of its water moiety (Casanueva et al. 2010; Klähn and Hagemann 2011). In addition, further strategies applied by cold-adapted organisms include ice-nucleating and anti-freeze proteins (Wilson and Walker 2010), recycling of nucleotides and peptides (Bergholz et al. 2009), and changing metabolic pathways (Sardesai and Babu 2000).

To date, only a few species have been identified that are both psychrophilic and acidophilic. One example is *Acidithiobacillus ferrivorans* (originally described as *Acidithiobacillus ferrooxidans* strain SS3) that was first isolated in Siberia (Kupka et al. 2007). While it shows its fastest growth rate at ~22 °C, it is capable of growing at temperatures as low as 5 °C (Hallberg et al. 2009)*. At. ferrivorans* oxidizes both iron (Kupka et al. 2007) and sulfur (Kupka et al. 2009), making it capable of catalyzing biomining at low temperatures (Dopson et al. 2007). Two *At. ferrivorans* strains have been genome sequenced (Liljeqvist et al. 2011; Talla et al. 2013) and potential genes involved in iron and ISC oxidation have been identified (Liljeqvist et al. 2013). Transcriptomes analyses were used to identify the expression of *At. ferrivorans* SS3 genes predicted to encode proteins for metabolism of ISC compounds such as Hdr and Sor that both oxidize elemental sulfur (Christel et al. 2016). In addition, a metagenomic study identified potential adaptations of acidophiles (including an *At. ferrivorans*-like population) to low temperatures in an acid mine drainage stream community in northern Sweden (Liljeqvist et al. 2015). These adaptations include CSPs, several pathways for the production of compatible solutes, growth as a biofilm, and an anti-freeze protein.

Little is known about the level of stress that is induced upon *At. ferrivorans* SS3 by culture at the two extremes of its growth temperature spectrum, and the adaptation strategies it employs. Therefore, this study aims to elucidate the *At. ferrivorans* transcriptional response to cultivation at 8 °C, a temperature relevant in low temperature mining environments (Liljeqvist et al. 2015), compared 20 °C, a temperature near to where it shows the highest growth rate.

## Materials and methods

### Strain and culturing

*At. ferrivorans* strain SS3 (DSM 17398) was pre-grown at 20 ± 2 °C in pH 2.5 mineral salts medium with trace elements and 50 mM ferrous iron as previously described (Christel et al. 2016). The culture was then inoculated into two separate 500 mL working volume continuous cultures (dilution rate of 0.03 L/day) with 5 mM potassium tetrathionate as electron donor (Christel et al. 2016). The continuous cultures were maintained at 20 ± 2 (the strains optimal temperature) and at 8 ± 1 °C (designated as low temperature). 8 ± 1 °C was chosen as it is a temperature typically encountered in, e.g., acid mine drainage streams in the active Kristineberg mine, Sweden, and an abandoned mine, Wales, in which *At. ferrivorans*-like populations were found to be present (Kay et al. 2013; Liljeqvist et al. 2015). The continuous cultures were confirmed as substrate limited by measurement of the tetrathionate concentration (Dopson and Lindström 1999).

### RNA extraction and sequencing

Duplicate 50 mL samples were taken from each of the continuous cultures representing growth at 20 and 8 °C. Nucleic acid extraction and RNA transcript sequencing were carried out as previously described (Christel et al. 2016). Briefly, the samples were rapidly cooled, pelleted, and the cells lysed. The lysate was cleaned and nucleic acids precipitated before genomic DNA was removed using the Turbo-DNA-free Kit (Ambion). Total RNA libraries (without ribosomal depletion) were prepared by SciLifeLab, Stockholm, Sweden using the Illumina TruSeq RNA Library Preparation Kit. RNA transcripts were sequenced at SciLifeLab on an Illumina HiSeq 2500 sequencer in high output mode (2 × 125 bp). Sequencing data are available from Figshare (https://dx.doi.org/10.6084/m9.figshare.2062656.v1).

### Bioinformatics

Bioinformatic analysis was essentially carried out as described in Christel et al. (2016) except that the “Tuxedo” pipeline was used for differential expression analysis and statistical calculation of RNA ‘Fragments per kilobase of exon per million fragments mapped’ (FPKM) with 95 % confidence intervals (Trapnell et al. 2012). Transcript assembly and annotation was carried out using the *At. ferrivorans* genome as reference (Refseq NC_015942.1; Liljeqvist et al. 2011). Hidden Markov models of selected proteins (e.g. trehalose synthesis pathways) were obtained from PFAM (Finn et al. 2015) and compared to the genome and transcripts using HMMER (3.1b2). Genes with significantly different transcript counts were classified using the SEED database (Overbeek et al. 2004).

## Results and discussion

*At. ferrivorans* SS3 was successfully grown in steady-state continuous culture at 20 and 8 °C, confirming its previously reported growth in cold environments (Hallberg et al. 2009).

### Identification of potential *At. ferrivorans* SS3 adaptations to low temperature

The *At. ferrivorans* reference genome contains several potential adaptations to a cold life-style that have been previously described in the literature, including genes coding for (a) CSPs; (b) molecular chaperones and helicases; (c) cell wall and membrane modification; (d) compatible solute production and transport; (e) oxidative stress regulation; and (f) biofilm formation (Table 1).

**Table 1:** *At. ferrivorans* genes predicted to be associated with growth at low temperature and their RNA transcript response to growth at optimal and low temperatures

Gene^a^	Function	FPKM^b^		Significance^c^
		20 °C	8 °C	
Cold shock proteins				
Acife_0085	Cold shock protein (DNA-binding domain)	671	6397	NS
Acife_0976	Cold shock protein (DNA-binding domain)	4563	14046	NS
Acife_2932	Cold shock protein (DNA-binding domain)	3880	45648	+
Chaperones and helicases				
Acife_0366	DEAD/DEAH box helicase	255	209	NS
Acife_0675	DEAD/DEAH box helicase	194	219	NS
Acife_0883	DEAD/DEAH box helicase	39	112	NS
Acife_1400	Trigger factor TF	71528	213237	NS
Acife_1953	DEAD/DEAH box helicase	829	1339	NS
Acife_2282	Molecular chaperone GroES	1558	549	–
Acife_2283	Molecular chaperone GroEL	1558	549	–
Acife_2914	Survival protein SurA	31	44	NS
Acife_3104	DNA helicase II	19	2	–
	10 × molecular chaperone^d^			NS
	17 × helicase (various)^d^			NS
Cell wall and membrane modification				
Acife_0117	Lipid A biosynthesis acyltransferase	124	18	NS
Acife_1274	Hopanoid biosynthesis associated radical SAM protein HpnJ	613	1228	NS
Acife_1275	Hopanoid biosynthesis associated protein HpnK	613	1228	NS
Acife_1278	Hopanoid biosynthesis associated radical SAM protein HpnH	613	1228	NS
Acife_1340	Methyltransferase	190	7	–
Acife_1348	Hopanoid biosynthesis associated membrane protein HpnM	65	188	NS
Acife_2401	Bactoprenol glucosyl transferase	7	62	+
Acife_1712	Fatty acid desaturase	124	376	NS
Acife_2790	Lipid A biosynthesis acyltransferase	11	2	NS
Acife_3203	Methyltransferase type 12	9	0	–
	72 × methyltransferase/methylase (various)^d^			
Compatible solutes and transport				
Acife_0606	Trehalose synthase (TS/TreS)	46	15	NS
Acife_0608	Malto-oligosyltrehalose trehalohydrolase (TreY)	46	15	NS
Acife_0609	4-alpha-glucanotransferase (TreZ)	46	15	NS
Acife_0928	ABC transporter ATP-binding protein	22	73	+
Acife_1168	Mannosyltransferase (TPS)	1572	1538	NS
Acife_1383	Glycine betaine transporter	314	4454	NS
Acife_1677	Sucrose-phosphate synthase	50	126	NS
Acife_1678	Sucrose synthase	50	126	NS
Acife_2037	Mechanosensitive ion channel protein	16	52	+
Acife_2898	PTS sugar transporter subunit	1558	760	NS
Acife_2901	PTS sugar transporter	1558	760	NS
	5 × ABC transporter (various)^d^			–
	28 × ABC transporter (various)^d^			NS
Oxidative stress response				
Acife_0490	Cobalt transporter	41	137	NS
Acife_1063	Cobalt transporter	190	7	–
Acife_1434	Superoxide dismutase	425	1322	NS
Acife_1891	Cobyrinic acid a,c-diamide synthase CbiA	149	61	NS
Acife_2081	Cobalamin biosynthesis protein CbiX	11	5	NS
Acife_2190	Cobalt transporter	27	13	NS
Acife_2431	Catalase	197	371	NS
Acife_2993	Cobalamin biosynthesis protein CobS	14	0	NS
Acife_3135	Cobyrinic acid a,c-diamide synthase CbiA	19	17	NS
	3 × peroxiredoxin^d^			NS
	3 × peroxidase (various)^d^			NS
	2 × hydroperoxide reductase^d^			NS
Biofilm formation				
Acife_0273	Type II and III secretion system protein	0	1	NS
Acife_1000	Colanic acid biosynthesis glycosyltransferase WcaL	21	2	–
Acife_2158	Pilus assembly protein PilM	263	817	+
Acife_2159	Pilus assembly protein PilN	263	817	+
Acife_2160	Pilus assembly protein PilO	263	817	+
Acife_2161	Pilus assembly protein PilP	263	817	+
Acife_2793	LPS heptosyltransferase	53	195	+
Acife_2794	LPS biosynthesis protein	53	195	+
Acife_2861	General secretory pathway protein GspE	876	631	NS
	5 × LPS related (various)^d^			NS
	3 × secretion protein/secretion system protein^d^			NS
	2 × type II secretion system protein E^d^			NS
	2 × type II secretion system protein F^d^			NS
	2 × type IV secretory pathway protein^d^			NS
	19 × pili related protein (various)^d^			NS

^a^Gene ID on the *At. ferrivorans* SS3 draft genome (RefSeq NC_015942.1)

^b^Fragments per kilobase of exon per million fragments mapped

^c^Significant at *p* ≤ 0.05; (+) and (−) denote significantly increased and decreased transcript counts during culture at 8 °C, respectively, while (NS) denotes no significant difference between the transcript counts

^d^See detailed information in Supplemental File 2

Three CSPs were present on the *At. ferrivorans* SS3 genome (Acife_0085, Acife_0976, and Acife_2932) that are predicted to be DNA-binding domain proteins that aid in unfolding the DNA double helix for enhanced transcription in the cold (Wistow 1990). In addition, the DNA-binding domain of these CSPs has a potential second function of binding and unfolding RNA (Landsman 1992).

A second potential cold adaptation strategy lies within the observed *At. ferrivorans* SS3 genes predicted to code for 13 molecular chaperones and 22 helicases of various kinds. Among these are four DEAD/DEAH helicases (Acife_0366, Acife_0675, Acife_0883, and Acife_1953) as well as trigger factor TF (Acife_1400). DEAD/DEAH helicases are nucleotide binding proteins up-regulated upon cold shock in *Psychrobacter arcticus* (Bergholz et al. 2009; Kuhn 2012) while trigger factor TF acts as a chaperone that accumulates during exposure to cold, enhancing cell viability (Kandror and Goldberg 1997). Additionally, the membrane associated chaperone survival protein SurA (Acife_2914) was encoded in the genome, potentially aiding the folding of outer membrane and excreted proteins (Lazar and Kolter 1996). Of special interest in this category were the chaperones GroES and GroEL (Acife_2282 and Acife_2283, respectively) as their expression is often altered during the bacterial cold response (Goodchild et al. 2004; Zheng et al. 2007), although the direction of the regulation as well as its extent varies (Yoshimune et al. 2013).

Cell wall and membrane modifications often play a major role in adaptation to cold. While genes such as bactoprenol glucosyl transferase (Acife_2041) can be used to influence the cell wall in *At. ferrivorans* SS3, they also exhibit a fatty acid desaturase (Acife_1712), used to maintain membrane fluidity by introducing double bonds into the acyl chains of lipids (Chintalapati et al. 2004). To exchange the fatty acid chains of already incorporated lipids, two copies of lipid A biosynthesis acyltransferase (Acife_0117 and Acife_2790) are available (Vorachek-Warren et al. 2002). Additionally, the membrane can regain lost fluidity by methylation of the lipid’s head group (Chintalapati et al. 2004). Seventy-four different methyltransferases and methylases were found in the *At. ferrivorans* SS3 genome of which, several could potentially fulfill this function. Lastly, four genes involved in hopanoid biosynthesis were predicted (Acife_1274, Acife_1275, Acife_1278, and Acife_1348). The products of hopanoid biosynthesis are sterol-like lipids involved in the regulation of membrane fluidity and stability (Kannenberg and Poralla 1999).

Another cold adaptation strategy coded within the genome is the biosynthesis and assimilation of compatible solutes. Thirty-three unspecified ABC transporters, a mechanosensitive ion channel protein (Acife_2037), glycine betaine transporter (Acife_1383), and PTS sugar transporters (Acife_2898, Acife_2901) could potentially introduce osmo- and cryoprotectants into the cell (Pflüger and Müller 2004). Additionally, sucrose synthase and sucrose-phosphate synthase (Acife_1677 and Acife_1678) may synthesize compatible solutes found in cold-grown cyanobacteria (Klähn and Hagemann 2011). Lastly, several genes involved in production of the cryoprotectant trehalose have been identified (Schade et al. 2004; Liljeqvist et al. 2015) including mannosyltransferase (TPS; Acife_1168), trehalose synthase (TreS; Acife_0606), maltooligosyltrehalose trehalohydrolase (TreY; Acife_0608), and 4-alpha-glucanotransferase (TreZ; Acife_0609).

Due to the higher solubility of gases at low temperature, the production of reactive oxygen species (ROS) is a major threat to organisms in low temperature environments. In the search for ROS scavenging and similar proteins, genes predicted to code for cobalamin biosynthesis were identified. Cobalamin is a cobalt-coordinated tetra pyrrole and is used as an enzyme cofactor in prokaryotes. However, it has also been reported to restore the activity of other ROS scavenging proteins (Majumdar et al. 2012; Chattopadhyay et al. 2012) that were found in the genome, namely catalase (Acife_2431) and superoxide dismutase (Acife_1434). Its genomic production potentially includes three cobalt transporters (Acife_0490, Acife_1063, and Acife_2190), as well as the cobalamin biosynthesis proteins CobS (Acife_2993), CbiX (Acife_2081), and two copies of CbiA (Acife_1891 and Acife_3125). In addition, several other enzymes dealing with oxidative stress were found, including various peroxidases, peroxiredoxins, and hydroperoxide reductases.

Finally, genes related to biofilms were identified, a growth strategy reported to be induced by cold (Mancuso Nichols et al. 2005). Several genes predicted to code for proteins of two secretion systems (type II and type IV) were found as well as three secretion system proteins and the general secretory pathway protein GspE (Acife_2861). These proteins can be used to excrete extracellular polymeric substances, which form the matrix of a biofilm, and can aid in trapping liquid water and nutrients at low temperatures (Mancuso Nichols et al. 2005). Furthermore, seven genes connected to lipopolysaccharide (LPS) production and export were found. In addition to its function in protection from chemical agents as well as aiding cellular stability (Erridge et al. 2002), LPS further takes part in cell adhesion to surfaces (Abu-Lail and Camesano 2003; Li et al. 2014). Cell adhesion is also greatly enhanced by colonic acid (Hanna et al. 2003; Yoshida et al. 2015). As part of its biosynthesis pathway, a gene cluster containing colonic acid biosynthesis glycosyltransferase WcaL (Acife_1000) was detected in the genome. Lastly contributing to biofilm formation, more than 20 genes involved in pili formation were present. Pili are suggested to play roles in initial cell attachment to abiotic surfaces, e.g., by *Escherichia coli* (Pratt and Kolter 1998), but also during development and maturation of other bacterial biofilms (O’Toole and Kolter 1998; Konto-Ghiorghi et al. 2009). In *At. ferrivorans*, the cluster PilMNOP (Acife_2158–Acife_2161) was detected alongside copies of the proteins PilTVWZ, and CpaB.

### RNA transcript data

RNA sequencing produced on average 42.2 million reads per sample (range 38.0–44.1 million) with an aggregated percentage of bases that have quality score higher than Q30 of >95.36 % and a PhiX error rate of 0.34 (Supplemental File 1). To ensure quality, on average 8.5 % (range 6.16–10.99 %) of single stranded reads were discarded. The remaining sequences were aligned and mapped against the *At. ferrivorans* SS3 reference genome with a mean mapping efficiency of 97.4 % (range 96.2–98.2 %) while 63.0 % (range 52.7–69.8 %) were aligned to multiple locations of the genome.

**Fig. 1:**
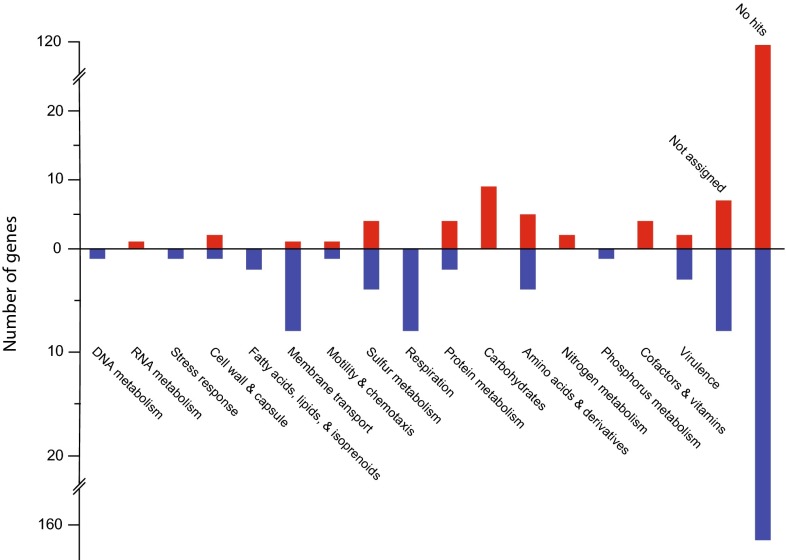
Number of genes as classified in the SEED database with significantly increased RNA transcript counts at 20 °C (*red*) and 8 °C (*blue*)

The *At. ferrivorans* SS3 response to growth at 8 °C compared to 20 °C resulted in 373 genes with significantly different RNA transcript counts, of which 213 were higher at 8 °C and 160 were higher at 20 °C (Supplemental File 2). Classification of these genes using the SEED database resulted in 287 genes without a match in the database (169 had higher counts at 8 °C and 118 at 20 °C) and an additional eight and seven genes were unable to be assigned to a class at 8 and 20 °C, respectively. The remaining 71 genes were sorted into 16 categories (Fig. 1). During growth at 8 °C, *At. ferrivorans* had a greater number of genes with higher RNA transcript counts for categories including energy production via sulfur metabolism and respiration; fatty acids and other membrane compounds; membrane transport; and the stress response. In contrast, categorized genes with higher transcript counts during growth at 20 °C mostly belonged to protein and carbohydrate metabolism, as well as cofactors and vitamin-related functions. Interestingly, different genes involved in the cell wall and capsule had higher RNA transcript levels at 8 and 20 °C, presumably due to changes in its structure due to the growth temperature.

### Analysis of cold adaptation-associated RNA transcripts during growth at 8 °C

A comparison of the 373 genes exhibiting significantly different RNA transcript counts with the *At. ferrivorans* genes suggested to be associated with cold adaptation (see the previous sections) gave 22 genes previously described to have a role in growth at low temperatures (Table 1). These 22 genes with significantly different RNA transcript counts constituted 13.8 % of the total cold adaptation predicted genes from the *At. ferrivorans* genome. In detail, of the three identified CSP genes, only Acife_2932 had a significantly greater number of RNA transcripts during growth at 8 °C (11.8-fold; FPKM 3880 to 45648). The increased number of Acife_2932 RNA transcripts supported its role as a cold-inducible protein as it had higher RNA transcripts during steady-state growth at low temperature. Potential reasons for the lack of response of the other two CSPs could be that Acife_0085 and Acife_0976 are purely cold shock-related genes and were not expressed during prolonged exposure to cold, that they have other functions than those related to growth at low temperature, or that the cultivation temperature of 8 °C was not low enough for an increase of expression of these two CSPs to be required.

Similar results were obtained when comparing RNA transcript counts of the chaperone and helicase genes. While many of these proteins are reported to be up-regulated during growth at low temperature (Barria et al. 2013), DNA helicase II (Acife_3104) had low FPKM values at both growth temperatures and had a 9.5-fold decreased RNA transcript count (FPKM 19–2), and no genes of this category exhibited increased counts at a lower growth temperature. This is in contrast to a previous study in which TF and SurA are found in higher concentrations in a cold-grown *Acidithiobacillus* strain (Mykytczuk et al. 2011). It is unclear if the difference between gene expression data in this study and protein amount as reported by Mykytczuk et al. was the result of differences in adaptation to cold or post-transcriptional regulation of this protein. However, the variance in regulation direction strongly suggests an adaptation difference between *At. ferrivorans* SS3 and the previously studied *Acidithiobacillus* strain. Two chaperones, GroES and GroEL (Acife_2282 and Acife_2283), had 2.8 fold lower RNA transcript counts in the cold (FPKM 1558–549), which is also in contrast to the previous proteomics study investigating cold adaptation (Mykytczuk et al. 2011). The lower number of GroES and GroEL RNA transcripts may be due to their having a role in response to heat rather than cold, and that *At. ferrivorans* SS3 was not under cold stress during growth at 8 °C, but rather heat stress at 20 °C.

Neither the fatty acid desaturase nor lipid A acyltransferase genes had higher RNA transcript counts at 8 °C. In addition, with the exception of a methyltransferase (Acife_1340) and methyltransferase type 12 (Acife_3203), the methylation systems detected in the genome were also largely unaffected. These two proteins showed lower RNA transcript counts (FPKM 190–7 and 9–0, respectively) despite that increased methylation of lipids is beneficial under cold conditions (Chintalapati et al. 2004). RNA transcript counts for genes predicted to be involved in hopanoid biosynthesis were not significantly different between the two growth temperatures. In contrast, the co-transcribed LPS-related genes heptosyltransferase (Acife_2793) and LPS biosynthesis protein (Acife_2794) exhibited a 3.7-fold increase in transcripts (FPKM 53–195), possibly affecting the stability of the outer membrane in cold conditions. However, increased production of LPS would likely increase the stability of the membrane at low temperature. Also, bactoprenol glucosyl transferase (Acife_2041) had 8.9-fold more RNA transcripts at low temperature (FPKM 7–62). Bactoprenol is responsible for the trans-membrane transport of the basic building blocks of the cell wall (Lovering et al. 2012). It has been previously proposed that a decrease of the bactoprenol pool via glycosylation can have regulatory effects (Inoue et al. 2013) and it is possible that *At. ferrivorans* SS3 utilizes this system to regulate the synthesis of new peptidoglycan layer components to compensate for the higher maintenance energy required for growth under cold conditions.

None of the genes predicted to code for the uptake or production of the compatible solutes glycine betaine, sucrose, or trehalose had different RNA transcript levels. Once again, this supports that *At. ferrivorans* SS3 is adapted to growth at low temperature. In contrast, a mechanosensitive ion channel protein (Acife_2037) had a 3.3-fold higher RNA transcript count (FPKM 16–52). Proteins of this type are able to sense stretching of the cell membrane, e.g. caused by hypo-osmotic shock, and act as an emergency valve to release compatible solutes and adjust cytosolic osmolarity (Haswell et al. 2011). As such, the increased number of RNA transcripts for this gene could be a measure of caution to prevent hypo-osmosis induced lysis of *At. ferrivorans* SS3 in conditions with rapidly changing salinity, i.e. influx of melt water.

No genes predicted to combat oxidative stress had significantly different levels of RNA transcripts. This was also largely true for the ROS scavenger restoring cobalamin biosynthesis pathway for which only one of the three cobalt transporters (Acife_1063) had 27.1-fold lower RNA transcript level (FPKM 190–7) at low temperature.

Several genes related to pili production seemed to be induced, with the PilMNOP operon (Acife_2158–Acife_2161) having 3.1-fold higher RNA transcripts (FPKM 263–817). Together with the increased LPS-related transcripts mentioned above, this regulatory response could indicate initiation of biofilm formation, starting with the production of compounds used for initial attachment to abiotic surfaces. Considering the minimal response of the systems reported above and the resulting conclusion that *At. ferrivorans* is adapted to culture at 8 °C, this could indicate that attachment to surfaces and subsequent biofilm formation is the first response of *At. ferrivorans* SS3 to growth at low temperature. However, in conflict with this hypothesis, adhesion enhancing colanic acid biosynthesis, represented by a co-transcribed gene cluster (Acife_0999–Acife_1003) had lower RNA transcript levels (FPKM 21–2).

### Analysis of other RNA transcripts with increased counts at low temperature

**Fig. 2:**
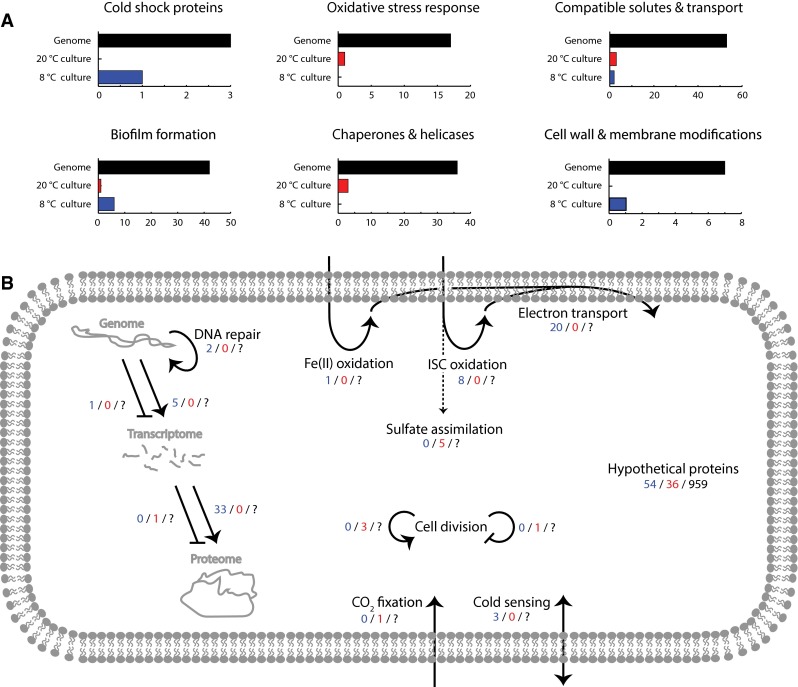
Response of known cold adaptation systems present on the *At. ferrivorans* SS3 genome via RNA transcript counts during culture at 8 °C compared to 20 °C (**a**) and a model of the cellular response to growth at 8 °C (**b**). Elements *colored in black* denote the total number of genes identified on the genome while *blue* and *red* denote genes with statistically valid increased RNA transcript counts at 8 and 20 °C, respectively

The majority of significant differentially expressed genes observed during the experiment had no immediate connection to growth at low temperature, or were insufficiently annotated (Supplemental File 2). *At. ferrivorans* SS3 seemed to adapt to the lower temperature by increasing transcript numbers related to energy metabolism, transcription, and translation (Fig. 2). In total, eight genes involved in ISC oxidation, 20 electron transport genes, and one gene involved in ferrous iron oxidation had higher RNA transcript counts. At the same time, three RNA polymerase subunits, 27 ribosomal proteins, and regulators and cofactors had higher numbers of RNA transcripts. Additionally, one ribosome silencing factor had lower counts. These results suggest that *At. ferrivorans* SS3 was not completely unaffected by reduced temperature as greater metabolic and electron transport proteins were required to maintain cell integrity and growth. In addition, more mRNAs were needed that possibly compensated for losses due to cold-induced mis-folding or oxidative damage. Another major *At. ferrivorans* SS3 response appeared to be DNA replication and damage as the co-transcribed DNA primase (Acife_2383) and DNA mismatch repair protein MutS showed a high 58.3-fold increase in transcript counts (FPKM 525–30598).

Systems with significantly lower transcript counts during cold growth include for example, the gene cluster FtsYEX (Acife_0556–Acife_0558; FPKM 246–41) related to cell division, and septum formation inhibitor Maf (Acife_0089; FPKM 111–13). Although all three copies of the carbon dioxide fixation protein RuBisCo present on the genome were expressed, only the large sub-unit RuBisCo (Acife_2232) had significantly lower transcript counts at low temperature (FPKM 502–33). In addition, a gene cluster responsible for sulfate assimilation had lower RNA transcript counts (Acife_2625–Acife_2629; FPKM 25–4), indicating decreased demand of the corresponding nutrient. Transcripts coding for bacterial RNase P (Acife_R0052) were also decreased (FPKM 43581–6760), conceivably to prevent wasteful destruction of repairable mRNAs. Lastly and as mentioned above, significant increased and decreased numbers of transcripts applied to 91 hypothetical genes with unknown functions.

### Regulation mechanisms relevant to culture at low temperature

Several regulatory systems commonly found in psychrophilic strains may have been responsible for the observed changes in transcript counts (Supplemental File 2). The change in temperature may have been sensed by three histidine kinases with greater RNA transcript counts (Sakamoto and Murata 2002). NusA (Acife_2651) is a transcription termination factor that had a 6.1-fold transcript count increase (FPKM 47–287) in the cold and is reported to be induced by CspA in *E. coli* (Bae et al. 2000). However, regulation of cold adaptation proteins can also occur post-transcriptionally (Giuliodori et al. 2004). In this study, transcripts coding for translation initiation factor IF3 (Acife_2538) and IF1 (Acife_2688) were 7.2- and 22.5-fold higher at 8 °C, respectively. These initiation factors are often observed to be up-regulated in cold-grown bacteria (Gualerzi et al. 2003) and have regulatory effects by stimulating preferential translation of cold shock genes (Weber and Marahiel 2003; Giuliodori et al. 2007; Barria et al. 2013). In addition to the initiation factors, transcript counts for elongation factor EF-Tu (Acife_2712) and EF-G (Acife_2713) were increased 7.5- and 6.0-fold, respectively (FPKM 70–523, and FPKM 108–648). Besides their main function as elongation factors, EF-Tu and EF-G are suggested to moonlight as chaperones (Caldas et al. 1998, 2000). Both genes are reported to be up-regulated in sub-zero growth of *Psychrobacter cryohalolentis* (Bakermans et al. 2007).

## Conclusions

The data obtained by this study suggests that *At. ferrivorans* SS3 does not exhibit a major cold stress response during culture at 8 °C (Fig. 2), a temperature typically encountered in cold mining environments. This suggests *At. ferrivorans* SS3 is adapted to growth at this temperature and should be classified as eurypsychrophile rather than psychrotolerant. Of 373 significantly differentially expressed genes, only 22 belong to previously reported adaptations to cold environments. Instead, *At. ferrivorans* copes with growth at 8 °C by reallocating efforts to increase energy metabolism plus mRNA and protein production.

## Electronic supplementary material

Below is the link to the electronic supplementary material.

Supplementary material 1 (PDF 285 kb)

## Abbreviations

ISCs: Inorganic sulfur compounds
CSP: Cold shock proteins
CIPs: Cold-inducible proteins
ROS: Reactive oxygen species
LPS: Lipopolysaccharide
FPKM: Fragments per kilobase of exon per million fragments mapped
