# Supplementary material for: Acidithiobacillus ferrivorans SS3 presents little RNA transcript response related to cold stress during growth at 8 °C suggesting it is a eurypsychrophile
Source: Extremophiles. 2016 Oct 25;20(6):903–13. doi: 10.1007/s00792-016-0882-2 (PMC5085989; doi:10.1007/s00792-016-0882-2)
Supplement: Supplementary file 1 — Supplementary material 1 (PDF 285 kb) [file 792_2016_882_MOESM1_ESM.pdf]

Acidithiobacillus ferrivorans SS3 presents little RNA transcript response related to cold stress during growth at 8 °C suggesting it is a eurypsychrophile

Stephan Christel, Jimmy Fridland, Elizabeth L. Watkin, & Mark Dopson

Supplemental Files

Supplemental File 1. RNA sequencing statistics

Replicate	Number of reads	PhiX error rate	>=Q30 (%)[a]
Optimum_101	38 048 551	0.34	95.47
Optimum_102	43 502 381	0.34	95.36
Cold_103	44 089 086	0.34	95.14
Cold_104	43 148 804	0.34	95.3

[a]Aggregated percentage of bases that have quality score more than Q30

Supplemental File 2. Full data table of significantly differentially expressed RNA transcripts from culture grown at 20° and 8°C.

			**20 °C**			**8 °C**				
**Gene[a]**	**Gene function**	**FPKM[b]**	**95% CI low**	**95% CI high**	**FPKM[b]**	**95% CI low**	**95% CI high**	**log2 (fold change)**	**Adjusted p-value (q)[c]**	**SEED classification[d]**
**Genes with higher transcript count at 8 °C**										
Acife_1561	hypothetical protein	0	0	0	909	543	1276	unique cold	0,0030	No hits
Acife_2355	NAD-dependent dehydratase	0	0	0	5	0	12	unique cold	0,0013	No hits
Acife_2356	ferredoxin	0	0	0	12	0	24	unique cold	0,0013	No hits
Acife_2359	NAD(P)H dehydrogenase (quinone)	0	0	0	4	0	11	unique cold	0,0469	No hits
Acife_R0051	tRNA	0	0	0	71606	0	153982	unique cold	0,0125	No hits
Acife_2378	hypothetical protein	525	0	2073	30598	27687	33508	5,86	0,0013	No hits
Acife_2379	hypothetical protein	525	0	2073	30598	27687	33508	5,86	0,0013	No hits
Acife_2381	integrase	525	0	2073	30598	27687	33508	5,86	0,0013	No hits
Acife_2382	RNA polymerase sigma factor RpoD	525	0	2073	30598	27687	33508	5,86	0,0013	No hits
Acife_2383	DNA primase	525	0	2073	30598	27687	33508	5,86	0,0013	No hits
Acife_2384	DNA mismatch repair protein MutS	525	0	2073	30598	27687	33508	5,86	0,0013	No hits
Acife_2385	aspartyl-tRNA amidotransferase subunit B	525	0	2073	30598	27687	33508	5,86	0,0013	No hits
Acife_2386	30S ribosomal protein S21	525	0	2073	30598	27687	33508	5,86	0,0013	No hits
Acife_2699	50S ribosomal protein L24	46	0	105	1919	621	3217	5,38	0,0013	No hits
Acife_3128	oxidoreductase	7	0	14	266	99	434	5,23	0,0013	No hits
Acife_1034	hypothetical protein	10	0	50	336	4	667	5,09	0,0369	No hits
Acife_2701	30S ribosomal protein S17	277	0	817	8107	0	17729	4,87	0,0030	No hits
Acife_2702	50S ribosomal protein L29	277	0	817	8107	0	17729	4,87	0,0030	No hits
Acife_2703	50S ribosomal protein L16	277	0	817	8107	0	17729	4,87	0,0030	No hits
Acife_2700	50S ribosomal protein L14	277	0	817	8107	0	17729	4,87	0,0030	Respiration
Acife_0893	hypothetical protein	3569	0	12081	94638	0	209497	4,73	0,0036	Amino Acids and Derivatives

Acife_0894	transporter	3569	0	12081	94638	0	209497	4,73	0,0036	Amino Acids and Derivatives
Acife_0895	RND transporter	3569	0	12081	94638	0	209497	4,73	0,0036	Amino Acids and Derivatives
Acife_0896	cation transporter	3569	0	12081	94638	0	209497	4,73	0,0036	Amino Acids and Derivatives
Acife_0899	MULTISPECIES: hypothetical protein	3569	0	12081	94638	0	209497	4,73	0,0036	No hits
Acife_0901	hypothetical protein	3569	0	12081	94638	0	209497	4,73	0,0036	No hits
Acife_0902	hypothetical protein	3569	0	12081	94638	0	209497	4,73	0,0036	No hits
Acife_0903	hypothetical protein	3569	0	12081	94638	0	209497	4,73	0,0036	No hits
Acife_0905	Fis family transcriptional regulator	3569	0	12081	94638	0	209497	4,73	0,0036	No hits
Acife_0906	hypothetical protein	3569	0	12081	94638	0	209497	4,73	0,0036	No hits
Acife_0897	MULTISPECIES: Ni/Fe hydrogenase	3569	0	12081	94638	0	209497	4,73	0,0036	Respiration
Acife_0898	cation transporter	3569	0	12081	94638	0	209497	4,73	0,0036	Virulence
Acife_2688	MULTISPECIES: translation initiation factor IF-1	125	0	332	2812	771	4854	4,49	0,0207	No hits
Acife_1847	disulfide isomerase	19	0	41	388	10	766	4,36	0,0036	Protein Metabolism
Acife_2698	50S ribosomal protein L5	92	16	167	1871	754	2989	4,35	0,0013	No hits
Acife_2669	phosphoserine phosphatase	8	0	18	146	130	162	4,14	0,0013	Amino Acids and Derivatives
Acife_2695	50S ribosomal protein L6	107	17	197	1826	657	2995	4,09	0,0013	No hits
Acife_2696	30S ribosomal protein S8	106	0	227	1408	489	2326	3,73	0,0013	No hits
Acife_2622	quinol oxidase	20	0	49	261	59	464	3,71	0,0013	No hits
Acife_1793	hypothetical protein	23	0	61	289	83	495	3,65	0,0425	No hits
Acife_2492	pseudo	416	65	767	4913	1774	8052	3,56	0,0013	Sulfur Metabolism
Acife_2932	cold-shock protein	3880	586	7174	45648	20787	70509	3,56	0,0013	No hits
Acife_2493	hypothetical protein	522	158	886	5954	2378	9529	3,51	0,0013	No hits
Acife_2708	50S ribosomal protein L23	156	0	369	1770	0	3674	3,50	0,0013	No hits
Acife_2709	50S ribosomal protein L4	156	0	369	1770	0	3674	3,50	0,0013	No hits
Acife_2440	hydrolase Nlp/P60	330	0	3824	3702	0	41399	3,49	0,0449	No hits
Acife_2441	hypothetical protein	330	0	3824	3702	0	41399	3,49	0,0449	No hits

Acife_2468	glycine cleavage system protein H	494	148	840	5478	2283	8673	3,47	0,0013	No hits
Acife_0107	hypothetical protein	8	1	16	91	26	155	3,45	0,0013	No hits
Acife_1221	glutathione S-transferase	5	0	12	58	20	97	3,44	0,0229	No hits
Acife_2838	dioxygenase	8	0	16	80	26	133	3,37	0,0013	No hits
Acife_3125	nitrogen regulatory protein P-II	46	0	156	474	0	1324	3,36	0,0262	No hits
Acife_3126	membrane protein	46	0	156	474	0	1324	3,36	0,0262	No hits
Acife_3127	hypothetical protein	46	0	156	474	0	1324	3,36	0,0262	Not assigned
Acife_2055	hypothetical protein	61	3	119	603	565	641	3,31	0,0117	No hits
Acife_2056	hypothetical protein	61	3	119	603	565	641	3,31	0,0117	Phosphorus Metabolism
Acife_2057	hypothetical protein	51	20	82	503	0	1752	3,30	0,0023	No hits
Acife_2058	hypothetical protein	51	20	82	503	0	1752	3,30	0,0023	No hits
Acife_2491	thiosulfate oxidation carrier complex protein SoxZ	1264	290	2238	11903	4591	19215	3,24	0,0013	Sulfur Metabolism
Acife_2723	preprotein translocase subunit SecE	102	0	235	953	282	1624	3,23	0,0145	No hits
Acife_R0053	rRNA	60057	1902	118212	543102	239221	846983	3,18	0,0013	No hits
Acife_0525	adenine methyltransferase	3	0	6	31	9	52	3,18	0,0013	DNA Metabolism
Acife_2041	bactoprenol glucosyl transferase	7	0	16	62	18	106	3,15	0,0232	No hits
Acife_2563	hypothetical protein	3	0	7	24	4	43	3,13	0,0353	No hits
Acife_2321	hypothetical protein	10	0	20	84	15	153	3,11	0,0049	Not assigned
Acife_0942	hypothetical protein	3	0	7	24	3	46	3,06	0,0391	No hits
Acife_2478	transcriptional regulator	314	9	620	2501	715	4287	2,99	0,0013	No hits
Acife_1329	dihydroorotate dehydrogenase	8	0	17	57	4	110	2,92	0,0333	Not assigned
Acife_2711	30S ribosomal protein S10	70	0	179	523	0	1127	2,89	0,0284	No hits
Acife_2712	elongation factor Tu	70	0	179	523	0	1127	2,89	0,0284	No hits
Acife_1331	hypothetical protein	37	0	83	270	59	481	2,85	0,0388	No hits
Acife_2538	translation initiation factor IF-3	205	57	352	1472	542	2402	2,85	0,0013	No hits
Acife_0946	diguanylate cyclase	24	1	47	167	42	291	2,78	0,0099	No hits

Acife_2717	DNA-directed RNA polymerase subunit beta	35	12	58	238	87	390	2,77	0,0013	No hits
Acife_2544	NADH-quinone oxidoreductase subunit K	47	0	101	313	91	535	2,75	0,0167	No hits
Acife_2545	NADH:ubiquinone oxidoreductase subunit J	47	0	101	313	91	535	2,75	0,0167	No hits
Acife_2689	adenylate kinase	574	0	1563	3822	0	16297	2,73	0,0013	No hits
Acife_2690	preprotein translocase subunit SecY	574	0	1563	3822	0	16297	2,73	0,0013	No hits
Acife_2691	50S ribosomal protein L15	574	0	1563	3822	0	16297	2,73	0,0013	No hits
Acife_2692	50S ribosomal protein L30	574	0	1563	3822	0	16297	2,73	0,0013	No hits
Acife_2693	30S ribosomal protein S5	574	0	1563	3822	0	16297	2,73	0,0013	No hits
Acife_2371	type II restriction endonuclease	20	1	40	133	36	229	2,71	0,0036	No hits
Acife_2372	ATPase AAA	20	1	40	133	36	229	2,71	0,0036	No hits
Acife_2373	hypothetical protein	20	1	40	133	36	229	2,71	0,0036	No hits
Acife_2510	GTP-binding protein	47	0	94	307	104	511	2,71	0,0043	No hits
Acife_2511	hypothetical protein	47	0	94	307	104	511	2,71	0,0043	No hits
Acife_2686	30S ribosomal protein S13	120	0	242	763	192	1333	2,67	0,0125	No hits
Acife_2494	thiosulfohydrolase SoxB	514	188	840	3270	1248	5292	2,67	0,0013	Sulfur Metabolism
Acife_2589	rhodanese-like protein	17	0	39	106	11	202	2,67	0,0428	No hits
Acife_2471	disulfide reductase	838	308	1368	5320	2111	8528	2,67	0,0013	No hits
Acife_2472	heterodisulfide reductase subunit C	838	308	1368	5320	2111	8528	2,67	0,0013	No hits
Acife_2490	thiosulfate oxidation carrier protein SoxY	1721	581	2861	10807	3661	17952	2,65	0,0013	Sulfur Metabolism
Acife_2652	ribosome maturation factor	68	0	137	429	133	724	2,65	0,0049	No hits
Acife_2651	transcription termination factor NusA	47	14	80	287	107	468	2,61	0,0013	No hits
Acife_2684	30S ribosomal protein S4	90	21	159	551	197	906	2,61	0,0013	No hits
Acife_3132	VWA domain-containing protein	7	1	14	45	12	78	2,60	0,0013	No hits
Acife_2713	elongation factor G	108	37	178	648	262	1033	2,59	0,0013	No hits
Acife_2069	cation-binding protein	33	0	71	197	53	340	2,58	0,0428	No hits

Acife_2070	glycine cleavage system protein H	33	0	71	197	53	340	2,58	0,0428	Not assigned
Acife_2725	membrane protein	303	93	514	1810	724	2896	2,58	0,0013	Motility and Chemotaxis
Acife_2370	hypothetical protein	4	0	8	24	8	41	2,57	0,0043	No hits
Acife_1378	hypothetical protein	64	10	118	373	140	605	2,55	0,0030	No hits
Acife_2721	50S ribosomal protein L11	210	47	373	1213	462	1964	2,53	0,0013	No hits
Acife_2566	hypothetical protein	16	1	30	90	21	158	2,52	0,0054	No hits
Acife_0717	AntA/AntB antirepressor domain-containing protein	23	0	55	134	0	340	2,52	0,0425	Membrane Transport
Acife_0719	TRAG family protein	23	0	55	134	0	340	2,52	0,0425	Membrane Transport
Acife_0718	hypothetical protein	23	0	55	134	0	340	2,52	0,0425	No hits
Acife_0720	HipA domain-containing protein	23	0	55	134	0	340	2,52	0,0425	No hits
Acife_0721	hypothetical protein	23	0	55	134	0	340	2,52	0,0425	No hits
Acife_1844	pseudo	75	17	132	419	81	756	2,49	0,0191	No hits
Acife_3012	hypothetical protein	87	0	276	484	0	1638	2,48	0,0403	Fatty Acids, Lipids, and Isoprenoids
Acife_3019	hypothetical protein	87	0	276	484	0	1638	2,48	0,0403	Fatty Acids, Lipids, and Isoprenoids
Acife_3011	hypothetical protein	87	0	276	484	0	1638	2,48	0,0403	No hits
Acife_3013	hypothetical protein	87	0	276	484	0	1638	2,48	0,0403	No hits
Acife_3014	membrane protein	87	0	276	484	0	1638	2,48	0,0403	No hits
Acife_3015	FAD-linked oxidase	87	0	276	484	0	1638	2,48	0,0403	No hits
Acife_3016	short-chain dehydrogenase	87	0	276	484	0	1638	2,48	0,0403	No hits
Acife_3017	hypothetical protein	87	0	276	484	0	1638	2,48	0,0403	No hits
Acife_3018	NAD-dependent epimerase	87	0	276	484	0	1638	2,48	0,0403	No hits
Acife_3020	membrane protein	87	0	276	484	0	1638	2,48	0,0403	No hits
Acife_3021	hypothetical protein	87	0	276	484	0	1638	2,48	0,0403	No hits
Acife_1444	acyl carrier protein	492	0	1033	2708	889	4527	2,46	0,0133	Cell Wall and Capsule
Acife_3136	hypothetical protein	136	0	299	749	0	1563	2,46	0,0244	No hits
Acife_3137	bacterioferritin	136	0	299	749	0	1563	2,46	0,0244	No hits

Acife_2008	sulfotransferase	11	0	23	60	21	100	2,43	0,0211	No hits
Acife_2706	30S ribosomal protein S19	143	14	272	762	129	1395	2,41	0,0250	No hits
Acife_2929	preprotein translocase subunit TatC	62	13	110	323	116	531	2,39	0,0023	Membrane Transport
Acife_3133	ATPase AAA	26	1	51	135	27	243	2,36	0,0083	No hits
Acife_2961	hypothetical protein	500	144	857	2490	662	4319	2,32	0,0013	No hits
Acife_2962	hypothetical protein	500	144	857	2490	662	4319	2,32	0,0013	No hits
Acife_3073	hypothetical protein	81	14	147	395	3	786	2,29	0,0248	No hits
Acife_1812	heme ABC transporter ATP-binding protein	17	3	31	80	27	133	2,21	0,0054	No hits
Acife_1021	hypothetical protein	574	0	1250	2623	0	5589	2,19	0,0083	No hits
Acife_1014	hypothetical protein	574	0	1250	2623	0	5589	2,19	0,0083	Respiration
Acife_1015	cytochrome o ubiquinol oxidase	574	0	1250	2623	0	5589	2,19	0,0083	Respiration
Acife_1016	cytochrome c oxidase subunit I	574	0	1250	2623	0	5589	2,19	0,0083	Respiration
Acife_1017	cytochrome o ubiquinol oxidase	574	0	1250	2623	0	5589	2,19	0,0083	Respiration
Acife_1018	cytochrome o ubiquinol oxidase	574	0	1250	2623	0	5589	2,19	0,0083	Respiration
Acife_1020	protoheme IX farnesyltransferase	574	0	1250	2623	0	5589	2,19	0,0083	Respiration
Acife_2647	30S ribosomal protein S15	372	9	735	1644	594	2694	2,14	0,0133	No hits
Acife_2915	metal-dependent hydrolase	22	1	43	96	27	166	2,14	0,0325	No hits
Acife_2719	50S ribosomal protein L10	82	14	150	361	100	622	2,14	0,0043	No hits
Acife_3184	protein-serine/threonine phosphatase	67	15	119	295	76	514	2,14	0,0049	No hits
Acife_3185	serine/threonine protein kinase	67	15	119	295	76	514	2,14	0,0049	No hits
Acife_1330	protein kinase	35	7	64	155	58	251	2,13	0,0060	No hits
Acife_2710	50S ribosomal protein L3	72	23	122	316	125	506	2,13	0,0013	No hits
Acife_1859	2OG-Fe(II) oxygenase	2746	0	10443	11953	0	35903	2,12	0,0496	No hits
Acife_1860	hypothetical protein	2746	0	10443	11953	0	35903	2,12	0,0496	No hits
Acife_1861	MFS transporter	2746	0	10443	11953	0	35903	2,12	0,0496	No hits

Acife_1862	protoheme IX farnesyltransferase	2746	0	10443	11953	0	35903	2,12	0,0496	No hits
Acife_1863	hypothetical protein	2746	0	10443	11953	0	35903	2,12	0,0496	No hits
Acife_1864	rusticyanin	2746	0	10443	11953	0	35903	2,12	0,0496	No hits
Acife_1866	cytochrome c oxidase, aa3-type subunit IV	2746	0	10443	11953	0	35903	2,12	0,0496	No hits
Acife_1867	cytochrome c oxidase subunit III	2746	0	10443	11953	0	35903	2,12	0,0496	No hits
Acife_1868	cytochrome c oxidase subunit I	2746	0	10443	11953	0	35903	2,12	0,0496	No hits
Acife_1869	cytochrome c oxidase subunit II	2746	0	10443	11953	0	35903	2,12	0,0496	No hits
Acife_1870	hypothetical protein	2746	0	10443	11953	0	35903	2,12	0,0496	No hits
Acife_1871	cytochrome c552	2746	0	10443	11953	0	35903	2,12	0,0496	No hits
Acife_1872	cytochrome c	2746	0	10443	11953	0	35903	2,12	0,0496	No hits
Acife_1873	membrane protein	2746	0	10443	11953	0	35903	2,12	0,0496	No hits
Acife_1874	hypothetical protein	2746	0	10443	11953	0	35903	2,12	0,0496	No hits
Acife_1875	hypothetical protein	2746	0	10443	11953	0	35903	2,12	0,0496	No hits
Acife_1876	NADH dehydrogenase	2746	0	10443	11953	0	35903	2,12	0,0496	No hits
Acife_1865	hypothetical protein	2746	0	10443	11953	0	35903	2,12	0,0496	Not assigned
Acife_2481	dimethyl sulfoxide reductase subunit C	15	6	24	64	21	107	2,11	0,0060	No hits
Acife_2966	hypothetical protein	214	36	393	922	325	1518	2,10	0,0060	No hits
Acife_2464	radical SAM protein	82	24	140	350	133	567	2,09	0,0013	No hits
Acife_2469	hypothetical protein	268	49	486	1093	393	1792	2,03	0,0078	No hits
Acife_2470	glycine cleavage system protein H	268	49	486	1093	393	1792	2,03	0,0078	No hits
Acife_2963	hypothetical protein	98	20	176	397	126	668	2,02	0,0078	No hits
Acife_2509	hisitidine kinase	31	5	57	122	48	196	1,96	0,0145	No hits
Acife_1560	hypothetical protein	33	1	65	128	35	221	1,95	0,0327	No hits
Acife_2597	cytochrome o ubiquinol oxidase	24	4	44	94	30	159	1,95	0,0117	No hits
Acife_2477	NADH dehydrogenase	674	219	1128	2598	984	4212	1,95	0,0013	No hits
Acife_2964	hypothetical protein	151	21	282	583	168	999	1,95	0,0076	No hits

Acife_2707	50S ribosomal protein L2	158	46	270	609	218	1000	1,94	0,0030	No hits
Acife_2930	glycosyl transferase family 1	17	0	36	66	17	116	1,93	0,0191	No hits
Acife_2931	hypothetical protein	17	0	36	66	17	116	1,93	0,0191	No hits
Acife_2476	heterodisulfide reductase subunit C	664	229	1099	2522	947	4096	1,93	0,0030	No hits
Acife_2467	radical SAM protein	70	18	122	261	92	431	1,90	0,0073	No hits
Acife_2685	30S ribosomal protein S11	262	60	464	967	283	1651	1,88	0,0173	No hits
Acife_2793	LPS heptosyltransferase	53	12	94	195	77	314	1,88	0,0133	No hits
Acife_2794	LPS biosynthesis protein	53	12	94	195	77	314	1,88	0,0133	No hits
Acife_2795	polymerase	53	12	94	195	77	314	1,88	0,0133	No hits
Acife_2796	glycosyl transferase	53	12	94	195	77	314	1,88	0,0133	No hits
Acife_2508	hypothetical protein	42	11	73	149	51	248	1,84	0,0078	No hits
Acife_2596	cytochrome o ubiquinol oxidase	51	19	83	180	80	280	1,83	0,0036	No hits
Acife_2683	DNA-directed RNA polymerase subunit alpha	126	38	213	439	144	735	1,81	0,0054	No hits
Acife_2066	globin	119	18	220	416	137	695	1,80	0,0136	Stress Response
Acife_2483	formate dehydrogenase	57	20	94	196	80	312	1,78	0,0030	No hits
Acife_2716	DNA-directed RNA polymerase subunit beta\'	54	19	89	180	60	300	1,73	0,0049	No hits
Acife_0926	lysine--tRNA ligase	22	5	39	73	25	120	1,71	0,0167	No hits
Acife_0927	cell division protein FtsX	22	5	39	73	25	120	1,71	0,0167	No hits
Acife_0928	ABC transporter ATP-binding protein	22	5	39	73	25	120	1,71	0,0167	No hits
Acife_0925	peptide chain release factor 2	22	5	39	73	25	120	1,71	0,0167	Protein Metabolism
Acife_2715	MULTISPECIES: 30S ribosomal protein S12	299	70	527	971	293	1649	1,70	0,0136	No hits
Acife_2482	ferredoxin	39	5	72	126	38	213	1,70	0,0250	No hits
Acife_0201	hypothetical protein	77	16	137	248	79	417	1,70	0,0262	No hits
Acife_2037	mechanosensitive ion channel protein	16	3	29	52	16	88	1,69	0,0262	Not assigned
Acife_2486	phosphonate ABC transporter substrate-binding protein	94	30	158	305	96	515	1,69	0,0099	Membrane Transport

Acife_2487	two-component sensor histidine kinase	94	30	158	305	96	515	1,69	0,0099	Virulence
Acife_2215	histidine kinase	137	39	235	435	160	710	1,67	0,0167	No hits
Acife_2601	sulfur oxidation protein	93	24	163	292	117	466	1,65	0,0225	No hits
Acife_2158	pilus assembly protein PilM	263	68	457	817	211	1423	1,64	0,0243	Membrane Transport
Acife_2159	pilus assembly protein PilN	263	68	457	817	211	1423	1,64	0,0243	Membrane Transport
Acife_2160	pilus assembly protein PilO	263	68	457	817	211	1423	1,64	0,0243	Membrane Transport
Acife_2161	pilus assembly protein PilP	263	68	457	817	211	1423	1,64	0,0243	Membrane Transport
Acife_2720	50S ribosomal protein L1	95	28	161	289	99	479	1,61	0,0113	No hits
Acife_2463	membrane protein	1749	651	2846	5309	2322	8295	1,60	0,0036	No hits
Acife_1451	pseudo	135	23	247	401	133	669	1,57	0,0491	No hits
Acife_1842	membrane protein	195	59	331	576	211	941	1,56	0,0122	Not assigned
Acife_2543	NADH:ubiquinone oxidoreductase subunit L	99	31	167	266	120	413	1,43	0,0351	No hits
Acife_R0014	tRNA	397440	155997	638882	1032000	546323	1517690	1,38	0,0207	Not assigned
**Genes with higher transcript count at 20 °C**										
Acife_0428	hypothetical protein	8	0	21	0	0	0	unique	0,0294	No hits
Acife_1899	hypothetical protein	7	0	15	0	0	0	unique	0,0023	No hits
Acife_1906	hypothetical protein	23	0	67	0	0	0	unique	0,0277	No hits
Acife_1933	hypothetical protein	24	0	63	0	0	0	unique	0,0391	No hits
Acife_1936	hypothetical protein	8	0	19	0	0	0	unique	0,0049	No hits
Acife_2608	integration host factor subunit alpha	32	0	84	0	0	0	unique	0,0294	No hits
Acife_2787	rRNA methylase	6	0	17	0	0	0	unique	0,0198	No hits
Acife_3054	HNH endonuclease	4	0	7	0	0	0	unique	0,0013	No hits
Acife_3189	F0F1 ATP synthase subunit epsilon	5	0	13	0	0	0	unique	0,0195	No hits
Acife_3202	lipoprotein	9	0	27	0	0	0	unique	0,0477	No hits
Acife_3203	methyltransferase type 12	9	0	27	0	0	0	unique	0,0477	No hits

Acife_2020	alpha-glucan phosphorylase	33	10	57	12	2	22	-1,50	0,0486	Carbohydrates
Acife_2281	alpha/beta hydrolase	1558	0	4340	549	0	1579	-1,50	0,0248	No hits
Acife_2282	molecular chaperone GroES	1558	0	4340	549	0	1579	-1,50	0,0248	Protein Metabolism
Acife_2283	molecular chaperone GroEL	1558	0	4340	549	0	1579	-1,50	0,0248	Protein Metabolism
Acife_1624	ABC transporter substrate-binding protein	72	17	128	23	4	42	-1,63	0,0391	No hits
Acife_1376	phage shock protein A	416	145	687	132	18	246	-1,65	0,0243	No hits
Acife_2277	branched chain amino acid aminotransferase	131	37	226	42	10	74	-1,65	0,0412	Amino Acids and Derivatives
Acife_2558	dihydropteroate synthase	461	160	761	145	46	244	-1,66	0,0043	No hits
Acife_2559	ATP-dependent metalloprotease	461	160	761	145	46	244	-1,66	0,0043	No hits
Acife_2955	hypothetical protein	93	26	160	27	1	54	-1,76	0,0444	No hits
Acife_2230	ATPase AAA	98	10	186	28	6	50	-1,82	0,0460	No hits
Acife_3086	30S ribosomal protein S9	247	72	421	69	0	144	-1,85	0,0491	No hits
Acife_2201	Motility protein FimV	105	38	172	29	7	51	-1,86	0,0076	No hits
Acife_2570	hypothetical protein	58	14	103	15	2	28	-1,98	0,0200	No hits
Acife_0055	NRAMP family metal ion transporter	79	25	133	20	3	36	-1,99	0,0076	Membrane Transport
Acife_2604	sodium/hydrogen exchanger	16	4	28	4	0	9	-2,00	0,0391	No hits
Acife_2191	CBS domain-containing protein	142	35	249	35	2	68	-2,02	0,0225	No hits
Acife_2556	triose-phosphate isomerase	589	206	972	143	41	245	-2,04	0,0013	No hits
Acife_3105	cytochrome c biogenesis protein	79	24	134	19	5	33	-2,05	0,0200	Protein Metabolism
Acife_2053	transporter	679	244	1114	162	36	289	-2,06	0,0030	No hits
Acife_2205	molybdenum cofactor guanylyltransferase	59	11	107	14	0	29	-2,07	0,0417	Cofactors, Vitamins, Prosthetic Groups, Pigments
Acife_2206	molybdopterin-guanine dinucleotide biosynthesis protein MobB	59	11	107	14	0	29	-2,07	0,0417	Cofactors, Vitamins, Prosthetic Groups, Pigments
Acife_2167	glutamate synthase	40	14	66	9	1	18	-2,10	0,0030	Nitrogen Metabolism

Acife_2125	DNA topoisomerase IV subunit B	29	8	50	7	0	14	-2,11	0,0391	No hits
Acife_0198	transporter	8	0	17	2	0	4	-2,12	0,0331	No hits
Acife_0199	RND transporter	8	0	17	2	0	4	-2,12	0,0331	No hits
Acife_0200	transporter	8	0	17	2	0	4	-2,12	0,0331	No hits
Acife_2555	preprotein translocase subunit SecG	241	44	439	55	0	112	-2,14	0,0391	No hits
Acife_2229	VWA domain-containing protein	44	7	81	10	2	18	-2,17	0,0078	No hits
Acife_1914	DNA-directed RNA polymerase sigma-70 factor	80	20	141	18	2	33	-2,19	0,0173	No hits
Acife_3076	glutathione synthase	216	46	386	47	9	84	-2,20	0,0054	No hits
Acife_3077	glutamate--cysteine ligase	216	46	386	47	9	84	-2,20	0,0054	No hits
Acife_1937	hypothetical protein	52	11	93	11	0	22	-2,22	0,0331	No hits
Acife_0916	family 2 glycosyl transferase	67	19	115	14	3	26	-2,22	0,0066	No hits
Acife_2276	glutamine-synthetase adenylyltransferase	7	1	12	1	0	3	-2,25	0,0351	Nitrogen Metabolism
Acife_2004	succinyldiaminopimelate transaminase	44	11	78	9	0	19	-2,26	0,0294	Amino Acids and Derivatives
Acife_1210	TetR family transcriptional regulator	237	71	403	46	8	85	-2,36	0,0023	No hits
Acife_1113	thiazole synthase	81	20	143	15	0	33	-2,40	0,0260	Cofactors, Vitamins, Prosthetic Groups, Pigments
Acife_1114	hypothetical protein	81	20	143	15	0	33	-2,40	0,0260	No hits
Acife_2170	acyl-phosphate glycerol 3-phosphate acyltransferase	1031	343	1718	195	37	352	-2,40	0,0013	No hits
Acife_2171	serine protease	1031	343	1718	195	37	352	-2,40	0,0013	No hits
Acife_2266	bifunctional riboflavin kinase/FMN adenylyltransferase	45	9	81	8	0	19	-2,45	0,0425	No hits
Acife_1268	glycerol-3-phosphate dehydrogenase	41	8	73	7	0	17	-2,46	0,0359	Carbohydrates
Acife_2009	phosphoenolpyruvate synthase	93	30	156	17	3	31	-2,47	0,0013	Carbohydrates

Acife_2010	2,3-bisphosphoglycerate-independent phosphoglycerate mutase	93	30	156	17	3	31	-2,47	0,0013	Carbohydrates
Acife_0096	peptide-methionine (S)-S-oxide reductase	201	0	439	36	0	93	-2,47	0,0291	No hits
Acife_0097	peptide-methionine (R)-S-oxide reductase	201	0	439	36	0	93	-2,47	0,0291	No hits
Acife_0098	hypothetical protein	201	0	439	36	0	93	-2,47	0,0291	No hits
Acife_2219	hypothetical protein	124	34	213	22	0	47	-2,51	0,0099	No hits
Acife_0914	hypothetical protein	1269	435	2103	220	77	362	-2,53	0,0013	Not assigned
Acife_2627	phosphoadenosine phosphosulfate reductase	25	2	47	4	0	9	-2,54	0,0253	No hits
Acife_2625	sulfite reductase subunit alpha	25	2	47	4	0	9	-2,54	0,0253	Sulfur Metabolism
Acife_2626	sulfite reductase	25	2	47	4	0	9	-2,54	0,0253	Sulfur Metabolism
Acife_2628	sulfate adenylyltransferase subunit 2	25	2	47	4	0	9	-2,54	0,0253	Sulfur Metabolism
Acife_2629	sulfate adenylyltransferase	25	2	47	4	0	9	-2,54	0,0253	Sulfur Metabolism
Acife_0220	argininosuccinate lyase	465	24	906	80	0	180	-2,55	0,0113	Amino Acids and Derivatives
Acife_0222	diaminopimelate decarboxylase	465	24	906	80	0	180	-2,55	0,0113	Amino Acids and Derivatives
Acife_0216	hypothetical protein	465	24	906	80	0	180	-2,55	0,0113	Carbohydrates
Acife_0223	glucose-6-phosphate isomerase	465	24	906	80	0	180	-2,55	0,0113	Carbohydrates
Acife_0224	peptidase M15	465	24	906	80	0	180	-2,55	0,0113	Cell Wall and Capsule
Acife_0217	DNA-binding response regulator	465	24	906	80	0	180	-2,55	0,0113	No hits
Acife_0218	two-component sensor histidine kinase	465	24	906	80	0	180	-2,55	0,0113	No hits
Acife_0221	hypothetical protein	465	24	906	80	0	180	-2,55	0,0113	No hits
Acife_1775	hypothetical protein	3262	1227	5296	556	166	947	-2,55	0,0013	Not assigned
Acife_2225	guanylate kinase	85	15	154	14	0	33	-2,57	0,0371	No hits
Acife_2138	transporter	5	0	11	1	0	2	-2,58	0,0351	No hits

Acife_2140	transporter	5	0	11	1	0	2	-2,58	0,0351	Virulence
Acife_0558	cell division protein FtsX	246	71	421	41	4	77	-2,59	0,0023	Motility and Chemotaxis
Acife_0556	signal recognition particle-docking protein FtsY	246	71	421	41	4	77	-2,59	0,0023	No hits
Acife_0557	cell division protein FtsE	246	71	421	41	4	77	-2,59	0,0023	Not assigned
Acife_3093	hypothetical protein	98	33	164	16	4	29	-2,61	0,0013	No hits
Acife_3041	conjugal transfer protein TraF	83	7	158	13	0	27	-2,68	0,0089	No hits
Acife_3042	hypothetical protein	83	7	158	13	0	27	-2,68	0,0089	No hits
Acife_3043	conjugal transfer protein TraC	83	7	158	13	0	27	-2,68	0,0089	No hits
Acife_3044	DSBA oxidoreductase	83	7	158	13	0	27	-2,68	0,0089	No hits
Acife_3045	type II secretion system protein E	83	7	158	13	0	27	-2,68	0,0089	No hits
Acife_3046	thiol:disulfide interchange protein	83	7	158	13	0	27	-2,68	0,0089	No hits
Acife_3047	conjugal transfer protein TraA	83	7	158	13	0	27	-2,68	0,0089	Not assigned
rnpB	bacterial RNase P	43581	15411	71750	6760	689	12830	-2,69	0,0013	Not assigned
Acife_2526	2-isopropylmalate synthase	22	5	40	3	0	7	-2,72	0,0109	No hits
Acife_2670	glutathione amide-dependent peroxidase	783	212	1354	115	28	202	-2,77	0,0013	No hits
Acife_2807	AMP-binding protein	24	6	42	4	0	8	-2,78	0,0262	Cofactors, Vitamins, Prosthetic Groups, Pigments
Acife_2127	transporter	18	0	37	3	0	6	-2,80	0,0078	No hits
Acife_2128	RND transporter	18	0	37	3	0	6	-2,80	0,0078	No hits
Acife_0981	hypothetical protein	351	83	620	48	0	100	-2,87	0,0076	No hits
Acife_2939	dihydroorotase	47	7	87	6	0	13	-2,89	0,0036	No hits
Acife_1551	hypothetical protein	70	2	138	9	1	18	-2,92	0,0030	No hits
Acife_1553	type II secretion system protein F	70	2	138	9	1	18	-2,92	0,0030	No hits
Acife_1552	type IV pilus modification protein PilV	70	2	138	9	1	18	-2,92	0,0030	Not assigned
Acife_3097	GTP-binding protein	102	28	176	13	0	27	-2,93	0,0054	Protein Metabolism
Acife_2874	arginine--tRNA ligase	28	7	49	4	0	8	-2,93	0,0149	No hits

Acife_0056	phage shock protein A	107	25	189	14	0	29	-2,93	0,0066	No hits
Acife_2944	hypothetical protein	48	11	85	6	0	15	-2,94	0,0109	No hits
Acife_0088	ribonuclease G	111	0	225	13	0	29	-3,04	0,0013	No hits
Acife_0089	septum formation inhibitor Maf	111	0	225	13	0	29	-3,04	0,0013	No hits
Acife_0090	hypothetical protein	111	0	225	13	0	29	-3,04	0,0013	No hits
Acife_0091	hypothetical protein	111	0	225	13	0	29	-3,04	0,0013	No hits
Acife_0092	23S rRNA (pseudouridine(1915)-N(3))-methyltransferase RlmH	111	0	225	13	0	29	-3,04	0,0013	No hits
Acife_0093	ribosome silencing factor RsfS	111	0	225	13	0	29	-3,04	0,0013	No hits
Acife_0094	nicotinate-nicotinamide nucleotide adenylyltransferase	111	0	225	13	0	29	-3,04	0,0013	No hits
Acife_0095	thiol:disulfide interchange protein	111	0	225	13	0	29	-3,04	0,0013	No hits
Acife_3085	N-acetyl-gamma-glutamyl-phosphate reductase	34	6	62	4	0	9	-3,09	0,0109	No hits
Acife_0913	hypothetical protein	367	48	686	42	0	91	-3,13	0,0247	No hits
Acife_0910	hypothetical protein	99	7	191	11	0	26	-3,17	0,0244	No hits
Acife_2226	hypothetical protein	31	4	58	3	0	8	-3,18	0,0224	No hits
Acife_3104	DNA helicase II	19	4	34	2	0	6	-3,22	0,0485	No hits
Acife_2228	hypothetical protein	328	84	572	34	0	73	-3,26	0,0023	No hits
Acife_1909	conjugal transfer protein TrbB	37	6	69	3	0	8	-3,43	0,0173	No hits
Acife_0915	GAF sensor-containing diguanylate cyclase/phosphodiesterase	33	11	56	3	0	6	-3,47	0,0013	No hits
Acife_1003	UDP-glucose dehydrogenase	21	4	38	2	0	5	-3,47	0,0076	Carbohydrates
Acife_1002	ABC transporter permease	21	4	38	2	0	5	-3,47	0,0076	Cell Wall and Capsule
Acife_0998	hypothetical protein	21	4	38	2	0	5	-3,47	0,0076	No hits
Acife_0999	glycosyl transferase	21	4	38	2	0	5	-3,47	0,0076	No hits
Acife_1000	colanic acid biosynthesis glycosyltransferase WcaL	21	4	38	2	0	5	-3,47	0,0076	No hits
Acife_1001	glycosyl transferase	21	4	38	2	0	5	-3,47	0,0076	No hits

Acife_2599	hypothetical protein	200	66	335	17	0	37	-3,56	0,0013	No hits
Acife_0560	hypothetical protein	2037	333	3741	146	16	277	-3,80	0,0013	No hits
Acife_0559	RNA polymerase factor sigma-32	2037	333	3741	146	16	277	-3,80	0,0013	RNA Metabolism
Acife_2018	aldolase	131	29	234	9	0	24	-3,84	0,0013	Carbohydrates
Acife_2019	hypothetical protein	131	29	234	9	0	24	-3,84	0,0013	Carbohydrates
Acife_2017	pyridine nucleotide-disulfide oxidoreductase	131	29	234	9	0	24	-3,84	0,0013	No hits
Acife_2232	ribulose bisophosphate carboxylase	502	88	916	33	0	67	-3,92	0,0013	No hits
Acife_2809	hypothetical protein	3092	1074	5110	201	30	373	-3,94	0,0013	No hits
Acife_1773	tRNA (guanine-N2)-dimethyltransferase	604	132	1076	33	0	69	-4,20	0,0013	No hits
Acife_1774	hypothetical protein	604	132	1076	33	0	69	-4,20	0,0013	No hits
Acife_0911	hypothetical protein	405	100	710	22	0	46	-4,20	0,0013	No hits
Acife_1343	MBL fold metallo-hydrolase	190	0	526	7	0	26	-4,72	0,0078	No hits
Acife_1344	transcriptional regulator	190	0	526	7	0	26	-4,72	0,0078	No hits
Acife_1345	MFS transporter	190	0	526	7	0	26	-4,72	0,0078	No hits
Acife_1340	methyltransferase	190	0	526	7	0	26	-4,72	0,0078	Virulence
Acife_1342	cobalt transporter	190	0	526	7	0	26	-4,72	0,0078	Virulence
Acife_0917	diguanylate phosphodiesterase	113	32	194	3	0	8	-5,03	0,0030	No hits
Acife_1557	pilin	1165	397	1933	32	0	66	-5,19	0,0013	Not assigned
Acife_2612	hypothetical protein	120	0	262	3	0	10	-5,27	0,0130	No hits
Acife_2613	hypothetical protein	120	0	262	3	0	10	-5,27	0,0130	No hits
Acife_2614	ErfK/YbiS/YcfS/YnhG family protein	120	0	262	3	0	10	-5,27	0,0130	No hits
Acife_0061	membrane protein	7774	7568	7979	166	0	415	-5,55	0,0013	No hits
Acife_0062	ABC transporter	7774	7568	7979	166	0	415	-5,55	0,0013	No hits
Acife_0063	ABC transporter permease	7774	7568	7979	166	0	415	-5,55	0,0013	No hits
Acife_0064	sulfonate ABC transporter substrate-binding protein	7774	7568	7979	166	0	415	-5,55	0,0013	No hits
Acife_0065	hypothetical protein	7774	7568	7979	166	0	415	-5,55	0,0013	No hits
Acife_0066	pyridine nucleotide-disulfide oxidoreductase	7774	7568	7979	166	0	415	-5,55	0,0013	No hits

Acife_0067	ferredoxin	7774	7568	7979	166	0	415	-5,55	0,0013	No hits
Acife_0068	PBS lyase	7774	7568	7979	166	0	415	-5,55	0,0013	No hits
Acife_0069	4Fe-4S ferredoxin iron-sulfur binding domain-containing protein	7774	7568	7979	166	0	415	-5,55	0,0013	No hits
Acife_0070	oxidoreductase	7774	7568	7979	166	0	415	-5,55	0,0013	No hits
Acife_0071	GntR family transcriptional regulator	7774	7568	7979	166	0	415	-5,55	0,0013	No hits
Acife_0072	coproporphyrinogen III oxidase	7774	7568	7979	166	0	415	-5,55	0,0013	No hits
Acife_0073	LysR family transcriptional regulator	7774	7568	7979	166	0	415	-5,55	0,0013	No hits

[a]Gene ID on the *At. ferrivorans* SS3 draft genome (RefSeq NC_015942.1)
[b]Fragments per kilobase of exon per million fragments mapped ± standard error from replicates
[c]False discovery rate (FDR) adjusted p-value
[d]Gene classification according to function predicted by SEED database
